# What should be done with antisocial personality disorder in the new edition of the diagnostic and statistical manual of mental disorders (DSM-V)?

**DOI:** 10.1186/1741-7015-8-66

**Published:** 2010-10-27

**Authors:** Morten Hesse

**Affiliations:** 1 Centre for Alcohol and Drug Research, University of Aarhus, Copenhagen, Denmark

## Abstract

Antisocial personality disorder, psychopathy, dissocial personality disorder and sociopathy are constructs that have generally been used to predict recidivism and dangerousness, alongside being used to exclude patients from treatment services. However, 'antisocial personality disorder' has recently begun to emerge as a treatment diagnosis, a development reflected within cognitive behaviour therapy and mentalisation-based psychotherapy. Many of the behaviour characteristics of antisocial personality disorder are, at the same time, being targeted by interventions at criminal justice settings. A significantly higher proportion of published articles focusing on antisocial personality concern treatment when compared to articles on psychopathy. Currently, the proposal for antisocial personality disorder for the Diagnostic and Statistical Manual of Mental Disorders, fifth edition, suggests a major change in the criteria for this disorder. While the present definition focuses mainly on observable behaviours, the proposed revision stresses interpersonal and emotional aspects of the disorder drawing on the concept of psychopathy. The present commentary suggests that developments leading to improvement in the diagnosis of this type of disorder should, rather than focusing exclusively on elements such as dangerousness and risk assessment, point us to ways in which patients can be treated for their problems.

## Introduction

The personality disorder currently known as antisocial personality disorder, as defined by the Diagnostic and Statistical Manual of Mental Disorders, fourth edition (DSM-IV), has a long history. It is a term often linked to Prichard's early 19th century concept of 'moral insanity' [1]. The term antisocial personality has been appearing in the DSM since its first edition in 1952, although it was initially labelled antisocial reaction under sociopathic personality disturbance [2].

Several related concepts describe the personality problems that can lead to antisocial behaviour. These concepts include psychopathy, dissocial personality disorder, and antisocial personality disorder [3]. In general, more than 50% of the variance of these constructs is shared (see, for example, [3,4]), although the concept of psychopathy encompasses a broader range of problems and behaviours compared to the other two [5].

Antisocial personality disorder is a disorder that is associated with substantial impairment of the individual [6]. Moreover, antisocial personality disorder has a negative impact on the people surrounding these individuals, including, for example, children growing up with a parent who has antisocial personality disorder [7], and spouses of people with antisocial personality disorder [8].

Given the impact that antisocial personality can have on individuals and society in general, one should expect that high priority be given to the development of effective treatments for this disorder. This is, however, not the case. Indeed, many therapists appear to reject patients with antisocial personality disorder [9]. Moreover, many antisocial personality disorder patients report that they do not believe that their personality is in need of change [10], and few treatments have actually been developed for the disorder [11].

The next version of the DSM, the DSM-V is currently in the pipeline. The key question now is, will the DSM-V foster more research into ways in which people with these types of problems can be helped or not?

The proposed criteria for antisocial personality disorder are known as 'antisocial/psychopathic prototype'. Like the widely used Psychopathy Checklist-Revised (PCL-R), this prototype encompasses antisocial behaviour, including aspects such as crime, interpersonal deficits, and callous-unemotional traits. What then would happen if the new criteria were to be introduced? The present commentary discusses the advantages and disadvantages of this development, in particular with a focus on the likelihood that the new diagnostic criteria will inspire the development of effective treatments.

## Discussion

Overall, mental illness should primarily be diagnosed so that patients can obtain optimal treatment. A secondary aim should be to safeguard society against dangerous individuals. For a disorder such as antisocial personality disorder, both aims are highly relevant. If patients diagnosed as having an antisocial personality disorder do not receive treatment, it may simply be because there is no way to help such patients, or because such patients are all 'treatment rejecting' [10].

Do we know for a fact that people with antisocial personality disorder cannot be treated? Experience with other types of psychiatric illness suggests that there are barriers to treatment, not all of which are intrinsic to the disorder itself. For example, the stigma attached to mental illness, the belief that available treatments will not be helpful, and the basic level of public awareness of the nature of mental illness, may all have an impact on the behaviour of both the patients and their family members, and also the behaviour of service providers (see, for example, [12]).

Few treatments have been tested for antisocial or psychopathic personality disorder. A review of psychosocial treatments for antisocial personality disorder found 11 studies in total [11]. However, only two of these studies can be considered to tailor specifically for antisocial personality disorder [13,14]. Nevertheless, findings reviewed across a range of areas revealed that patients with antisocial personality disorder are able to respond to treatment options designed to target a number of the symptoms associated with this condition [11]. These symptoms include substance abuse, driving under the influence, as well as anger and violence. Simultaneously developers of mentalisation-based treatment for borderline personality disorder are working to develop their treatment to be appropriate for patients with comorbid borderline and antisocial personality disorder [15].

Similarly, psychoeducational, group-based prison programmes have helped patients change their criminal ways of thinking, reducing the risk of recidivism in turn [16-18] and in general, correctional rehabilitation is showing effects of practical significance for criminal recidivism and offender functioning [19]. Therefore, while it is true that the antisocial personality disorder diagnosis can sometimes be used to refuse treatment to patients, clinical research is becoming progressively more interested in viewing this diagnosis as a target for treatment.

### What's in a name?

As mentioned above, one change in the proposed criteria is the introduction of the term 'psychopathic'. Another notable change is a higher focus on issues pertaining to interpersonal and emotional aspects of the psychopathology.

When it comes to the concept of psychopathy, the main focus of this concept has been on its utility in forensic settings, and very little has been concerned with the effective treatment of patients.

The concept of psychopathy is strongly related to predicting the risk of recidivism in criminal behaviour; 7 years ago, a meta-analysis of the single most important instrument used in psychopathy, the PCL-R, found 16 studies that had assessed the PCL-R as a predictor of institutional adjustment, and 34 studies that had assessed the PCL-R as a predictor of criminal recidivism [20]. In contrast, research addressing psychopathy as a target for treatment is far more limited.

Thus, while it has been claimed that psychological treatments for psychopathy are ineffective or may even worsen the outcome in patients, the evidence supporting this claim is limited [21]. Where is the evidence that suggests that psychopathic and antisocial patients cannot be treated? The evidence rests mainly on findings that indicate that psychopathy and, to a much lesser extent, antisocial personality, represent a negative prognostic factor in many contexts [5]. Conversely, although it has been noted that higher severity predicts poorer outcome, this does not mean that treatment cannot be of some benefit; as mentioned above, many of the problems characteristic of individuals with psychopathy and antisocial personality respond to such treatment. This leads to the question: what would happen in this line of research if the concept of psychopathy were to replace the concept of antisocial personality disorder? Would researchers and clinicians continue to refine tools for evaluating dangerousness, and would the focus on treatment and counselling slip out of focus?

A search on the PubMed database (conducted 15 August 2010) revealed that out of 1,678 hits on 'psychopathic personality' or 'psychopathy', a total of 393 articles contained words such as 'psychotherapy', 'counselling', or 'treatment' (23% of the articles). In comparison, 2,761 of a total of 7,299 hits (38%) on 'antisocial personality' contained the same words. While certainly not all of the 2,761 articles containing the word with 'antisocial personality' and mention of 'psychotherapy' or 'counselling' can be considered as representing important works that drive a treatment agenda forward, it seems to imply that the emerging interest in actually treating patients with antisocial disorder is borne by the label of 'antisocial', rather than the label of 'psychopathy'.

Indeed, in an article concerning a recent trial of cognitive behaviour therapy for antisocial personality disorder and violence, the authors stated their surprise that the patients with antisocial personality disorder were actually willing to enter treatment that was targeted for this disorder [13]. My colleagues and I have noted the same thing in an ongoing study taking place in substance abuse treatment settings in Denmark (unpublished results; Thylstrup and Hesse). Even in the absence of tangible rewards, it appears that a number of antisocial disorder patients are willing to try treatment and even attend sessions when they are presented with a careful description of their disorder and the problems that it causes, and offered a treatment that targets this disorder.

Like other socially disadvantaged patients, patients with antisocial personality disorder have problems with dropout rates and stability. However, psychiatric and substance abuse treatment services should not give up on the agenda of developing a range of treatments for antisocial behaviours. Psychotherapy will not turn serial killers or other extremely severe psychopaths into responsible citizens, and psychotherapy does not cure antisocial personality disorder. But many kinds of treatment can potentially be helpful for groups of patients whose behaviour is harming themselves and others.

### The significance of subtypes for treatment

Research into subtypes of psychopathy generally suggests that significant subtypes do exist. This research suggests that subtypes exist within the group of patients that have characteristics of psychopathy [22,23]. Some patients with high levels of psychopathy have a very low capacity for empathy, they experience low levels of anxiety and depression, score highly on traits such as callousness and narcissism and they have a strong tendency to use instrumental violence. Another group of patients with high levels of psychopathy experience high levels of symptoms such as anxiety and depression, as well as high comorbidity of conditions such as borderline personality disorder [24].

The latter group may be more responsive to treatment and it appears that they have the ability to form a working alliance with a therapist or counsellor [15,25]. Once again, this does not mean that we should exclude the more severely affected patients from treatment, it suggests that the development of effective treatments for psychopathy may best be preceded by targeting the patients who are comparatively easier to help, before progressing onto the more severely affected patients.

### What kind of treatment can be effective?

Wong and Hare have developed a set of guidelines listing potentially helpful treatments for psychopathy [26]. These guidelines may also apply to antisocial behaviour more generally. These guidelines include employing moral reasoning as an active part of treatment, using a cognitive behavioural approach, applying a social information processing approach, and planning for relapse prevention [26]. Additionally, one may add that treatment needs to structured, and that patients should not be required to address their emotional states. Asking the patients about 'feeling states' is unlikely to be helpful to those who have difficulty accessing such states, and who may act out aggressively when confronted with a potential personal shortcoming. And finally, a high level of external structure that may include supervision of the patient [27], as well as contingent reinforcement of specific prosocial behaviours [28], is likely to lead to improved outcomes in antisocial patients.

### What could go wrong in clinical practice?

There is another issue aside from research resources. The change in diagnostic criteria may also have an impact on clinical practice. The shift to describing antisocial behaviours within the broader concept of psychopathy can lead clinicians to make mistakes in two ways: the first is by failing to diagnose clinically relevant, potentially treatable psychopathology, because a patient with serious antisocial behaviour lacks additional features of psychopathy, such as callousness or failure to experience remorse. The second is by wrongly attributing these additional psychopathological features to patients who do not have them, thus overdiagnosing the disorder, but consequently failing to provide treatment, based on the false belief that all patients with antisocial personality are equally difficult to treat.

Therefore, the DSM-V should stress the interventions that antisocial patients may respond to, as well as the aspects of antisocial behaviours that may respond to treatment. Also, given the considerable evidence supporting the existence of clinically significant subtypes, the text should mention these subtypes, and mention that research indicating a very chronic and severe course of the disorder may not apply equally to all subtypes. While this is not standard in diagnoses, antisocial/psychopathic personality disorder is not a standard psychiatric disorder.

## Conclusions

Antisocial personality disorder is associated with suffering for the individual and, perhaps more so than any other psychiatric disorder, causes problems for people living with antisocial patients. Antisocial behaviours have been the target of several trials of psychosocial treatments, and although there is still substantial room for improvement, some of these treatments hold promise. The concept of psychopathy, however, has mainly been studied in relation to the prediction of negative outcomes. The DSM-V should provide clinicians with the tools to distinguish between patients with serious but potentially treatable behavioural problems among psychiatric patients and patients with substance use disorders, and patients whose pathology is more likely to be chronic and less likely to respond to treatment.

The diagnostic criteria should reflect the fact that some of the behavioural problems associated with antisocial personality disorder respond to treatment, that there is some evidence to support the effectiveness of treatments, and that, therefore, the agenda should remain on how to best help patients, not only produce arguments as to why they should be kept in prison and kept out of treatment.

## Competing interests

The author declares that they have no competing interests.

## Pre-publication history

The pre-publication history for this paper can be accessed here:

http://www.biomedcentral.com/1741-7015/8/66/prepub
